# Nanotechnology Approach for Exploring the Enhanced Bioactivities and Biochemical Characterization of Freshly Prepared *Nigella sativa* L. Nanosuspensions and Their Phytochemical Profile

**DOI:** 10.3389/fbioe.2022.888177

**Published:** 2022-05-17

**Authors:** Tayyab Ali, Fatma Hussain, Muhammad Naeem, Ajmal Khan, Ahmed Al-Harrasi

**Affiliations:** ^1^ Clinico-Molecular Biochemistry Laboratory, Department of Biochemistry, Faculty of Sciences, University of Agriculture, Faisalabad, Pakistan; ^2^ College of Life Science, Hebei Normal University, Shijiazhuang, China; ^3^ Natural and Medical Sciences Research Center, University of Nizwa, Nizwa, Oman

**Keywords:** *Nigella sativa*, nanosuspension, antiglycation, antioxidant, HPLC, FT-IR, GC

## Abstract

*Nigella sativa* is one of the most commonly used medicinal plants as it exhibits several pharmacological activities such as antioxidant, antibacterial, anticancer, antidiabetic, and hemolytic. The purpose of this study was to apply the nanotechnology approach for exploring the enhanced bioactivities of freshly prepared *Nigella sativa* L. nanosuspensions and the phytochemical profile of *N. sativa* seed ethanolic extract. In this study, we performed the biochemical characterization of *Nigella sativa* L. ethanolic extract through High-performance liquid chromatography (HPLC), Fourier-transform infrared spectroscopy (FT-IR), and Gas chromatography (GC), and bioactivities in terms of antioxidant, antidiabetic, antibacterial, and hemolytic activities of nanosuspension and extract were competitively studied. The results revealed that the nanosuspension of *N. sativa* seeds showed higher total phenolic (478.63 ± 5.00 mg GAE/100 g) and total flavonoid contents (192.23 ± 1.390 mg CE/100 g) than the ethanolic seed extract. The antioxidant activity was performed using the DPPH scavenging assay, and nanosuspension showed higher potential (16.74 ± 1.88%) than the extract. The antidiabetic activity was performed using antiglycation and α-amylase inhibition assays, nanosuspension showed higher antidiabetic potential [antiglycation (58 ± 0.912%)] and [bacterial α-amylase inhibition (18.0 ± 1.3675%)], respectively. Nanosuspension showed higher biofilm inhibition activity against *Escherichia coli* (66.44 ± 3.529%) than the extract (44.96 ± 2.238%) and ciprofloxacin (59.39 ± 3.013%). Hemolytic activity was performed and nanosuspension showed higher hemolytic activity than the extract as 7.8 ± 0.1% and 6.5 ± 0.3%, respectively. The study showed that nanosuspension had enhanced the bioavailability of bioactive plant compounds as compared to the ethanolic extract. Therefore, nanosuspension of *N. sativa* seed extract showed higher biochemical activities as compared to the ethanolic extract. This nanotechnology approach can be used as a platform for the development of combination protocols for the characterization of liquid state nanosuspensions in an adequate manner and also for therapeutic applications.

## 1 Introduction

Different drugs have been used for controlling the rate of infectious diseases. Excessive use of chemical-based drugs showed severe side effects on different tissues or organs. For example, acetaminophen causes liver cirrhosis, bromocriptine increases the risk of circulatory collapses, digoxin leads to severe gastrointestinal issues, and clarithromycin increases the risk of cardiovascular diseases. To overcome the risks and toxicity issues of these toxic drugs, medicinal plants have been used as they are a potential source of natural products with different perspectives; solubility and bioavailability have become vital roadblocks in developing novel pharmaceutical products (Yuan et al., 2016; Aslam et al., 2019). Medicinal plants exhibit a variety of natural compounds that increase their bioavailability, making them more valuable and safer for biological tissues. These compounds are antioxidants, anthocyanins, flavonoids, stilbenes, and phenolics (Yu et al., 2021). The natural extracts of plant species used worldwide for numerous purposes, including the cure of infectious diseases, are primarily due to the active compounds produced during the secondary metabolism (Jamshidi-Kia et al., 2018).

*Nigella sativa is* an annual blooming herb, a highly effective medicinal plant of the Ranunculaceae family. *N. sativa* is native to North Africa, Southwest Asia, and Southern Europe, referred to as black cumin or black seeds (Sharma et al., 2009). These *N. sativa* seeds are also used to cure different diseases, including hypertension, asthma, back pain, obesity, fever, dizziness, flu, cough, inflammation, and infections. Its extract and oil have been used as revolutionary healers and immunity boosters (Kazmi et al., 2019). *N. sativa* showed antitumor, serum-glucose lowering, smooth muscle relaxant, and anti-inflammatory properties. The extracts and different formulations of *N. sativa* have valuable effects on the hepatic, cardiovascular, pulmonary, gastrointestinal, renal, and central nervous system (Mohebbati and Abbasnezhad, 2020). The major bioactive compounds such as thymoquinone (30–48%), thymohydroquinone, dithymoquinone, cymene (7–15%), carvacrol (6–12%), 4-terpineol (2–7%), t-anethol (1–4%), -pinene, and thymol are all found in *N. sativa* oil (NSO) in significant amounts. The oil’s low toxicity implies a considerable margin of safety at therapeutic NSO levels (Sultan et al., 2022). Among all bioactive compounds, thymoquinone (TQ) is considered the most important medicinal component of *N. sativa* oil (NSO).

Different studies on the synthesis and formulations of nanosuspensions have been reported in some herbal plants. For example, *Silybum marianum*, *Elettaria cardamomum*, *Coriandrum sativum* (Jahan et al., 2016), *Terminalia arjuna* (Zafar et al., 2020), *Piper nigrum* (Zafar et al., 2019), *Ginkgo biloba* (Aslam et al., 2019), and *Allium cepa* (Zafar et al., 2022). However, little information is available on the formulations of novel nanosuspensions of *N. sativa*. Therefore, further studies are needed in order to explore the role of natural bioactive compounds in the nanosuspensions of *N. sativa.*

Some of the natural products that are found in medicinal plants needed to explore through nanotechnology for improving the methods for their extraction, purification, and bioavailability. Nanotechnology utilizes the different types of nanoparticles with smaller size and larger surface area than their bulk counterparts and possesses exceptional features, including chemical, optical, and thermal properties (Khan et al., 2019). As a result, nanomaterials have emerged as promising candidates for a variety of biological applications (Wei et al., 2019). Nanosuspension technology has been demonstrated to be a novel and profitable approach for increasing the bioavailability of poorly soluble medicines (Geetha et al., 2014). This approach is helpful for searching those natural products that are found in the medicinal plants and remain undiscovered (Wei et al., 2019).

Nanosuspensions have several unique qualities that make them more valuable for drug delivery. Through this approach, particle size significantly reduced, dissolving rate raised, and absorption rate increased. It can increase the bioavailability of the respective drugs. Nanosuspension can be made with composites that are not soluble in water but soluble in oil. The medicinal nanosuspension can be administered by topical, oral, parenteral, pulmonary, ocular, and other routes (Prabhakar and Bala Krishna, 2011). A wide range of NPs have recently been explored and examined for biomedical applications, emphasizing cancer treatments, nano-drug mediums against multi-drug resistant microbes, and nanoparticle-based antioxidant agents (Mosallam et al., 2018).

Keeping in view of the literature update, we hypothesized that nanosuspensions of *N. sativa* seed extract with enhanced bioactivities developed by nanoprecipitation nanotechnology approach may exhibit enhanced bioactivities as compared to its ethanolic extract concerning the major roadblock of enhanced bioavailability of therapeutic compounds. The formulations of *N. sativa* nanosuspensions lack necessary information for therapeutic applications. However, this type of approach was not reported in the literature before. The biochemical characterization of the compounds present in the ethanolic extract of *N. sativa* seeds was performed by Fourier-transform infrared spectroscopy (FTIR), High-performance liquid chromatography (HPLC), and Gas chromatography (GC), respectively. These cost-effective nanoformulations could serve as a platform for plant-based nanosuspensions.

## 2 Materials and Methods

### 2.1 Chemical and Reagents

All standards were obtained from Sigma-Aldrich Co. (St. Louis, United States) (phenolic acids: chlorogenic acid, gallic acid, HB acid, caffeic acid, vanillic acid, kaempherol, sinapic acid, ferulic acid, salicylic acid, coumarin, quercetin, benzoic acid, and rutin). The reagents of antioxidant activity were obtained from Sigma-Aldrich Co. (St. Louis, United States): Folin–Ciocalteu reagent, 2,2-diphenyl-1-picrylhydrazyl (DPPH). Polyvinyl alcohol (PVA) was obtained from Alpha-Aesar Co. (United States), and bovine serum albumin was obtained from Applichem Inc. (Germany). The solvents and reagents used in the HPLC analysis were purchased from Merck (Darmstadt, Germany). All chemicals and reagents used in the study were of analytical grade.

### 2.2 Collection of Plant Material and Extract Preparation

*Nigella sativa* (black cumin) seeds were collected randomly from the local registered market by following the guidelines from the Department of Botany, University of Agriculture Faisalabad. The seeds were dried and blended into a powder form and extraction was carried out using the Soxhlet apparatus (50 g of dried seeds powder and 500 ml of 95% ethanol as a solvent for 6 h). Then, the extract was double filtered using Whatman filter paper No. 1 and dried at room temperature (Mishra et al., 2013). The final obtained extract was stored in non-opaque sterilized glass bottles at −18°C.

### 2.3 Preparation of Nanosuspensions

**FIGURE 1:**
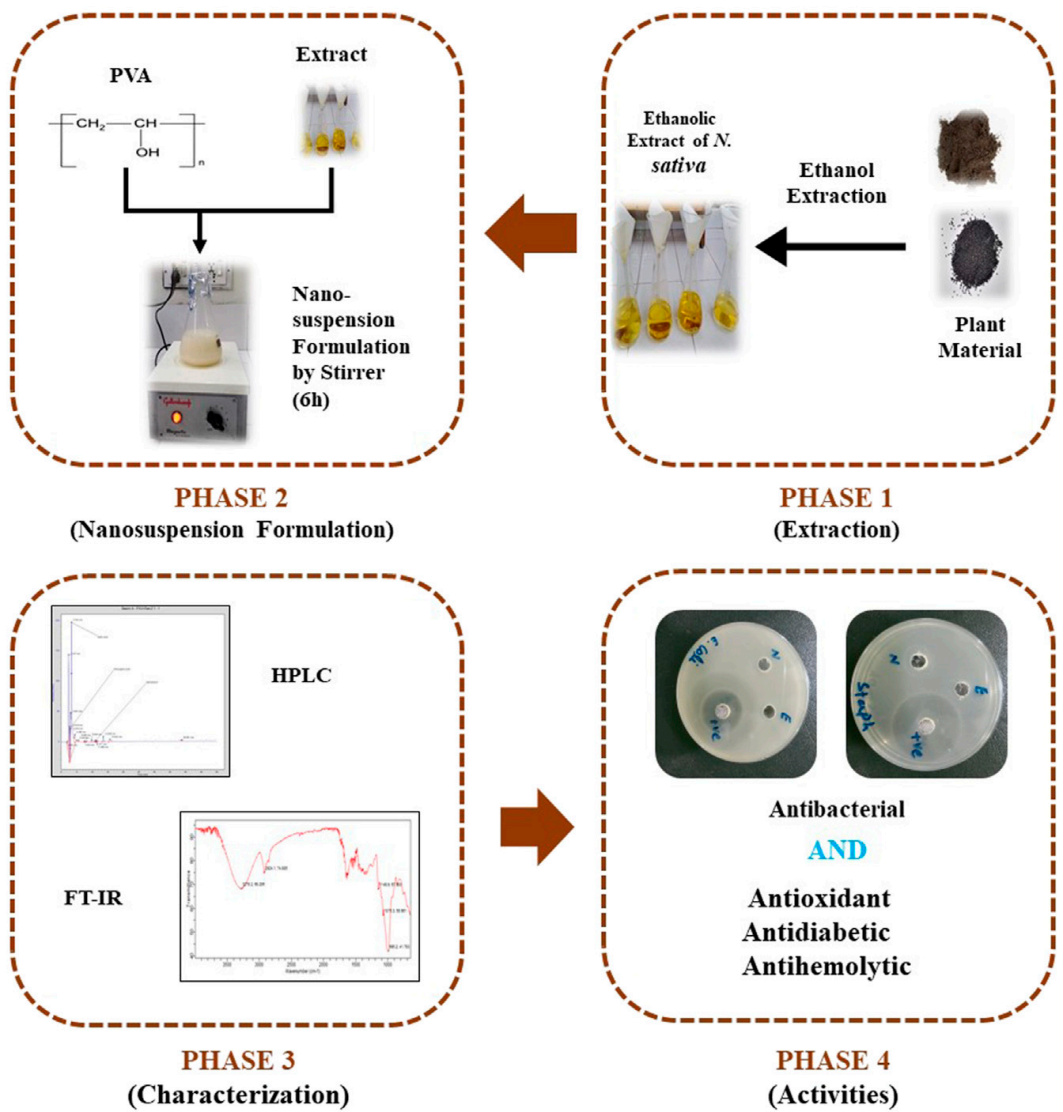
Principle phase of searching of bioactivities of *Nigella sativa* L. nanosuspension.

The nanosuspensions were prepared by following the nanoprecipitation method. Five gram of extract was dissolved into 30 ml of acetone and ethanol solution with a ratio (3:1) by adopting the protocols and precautionary measures. The solution was then gently injected into 50 ml of water containing 1.5 percent polyvinyl alcohol (PVA) by volume, with continuous magnetic stirring at 1,000 rpm. To avoid coalescence, the resultant emulsion was diluted in a 100 ml PVA solution (0.2 percent w/v in water) and stirred continuously at 500 rpm for 6 h at room temperature to allow solvent evaporation and nanosuspension generation. The resultant nanosuspension was then freeze-dried at −18°C (Figure 1) (Mishra et al., 2013).

### 2.4 Antioxidant Potential

#### 2.4.1 Total Phenolic Contents

The total Phenolic contents of *N. sativa* seed extract and nanosuspension were determined using the Folin–Ciocalteu method (Chahardehi et al., 2009). The reaction mixture was prepared by adding the 200 µL of 700 mM Na_2_CO_3_ solution, 250 µL of test samples, and 50 µL of Folin–Ciocalteu reagent (10%) and incubated at 25°C for 2 hours. The optical density (OD) was measured at 750 nm. Total phenolic contents in the extract and nanosuspension were calculated from the calibration curve of gallic acid.

#### 2.4.2 Total Flavonoid Contents

The total flavonoid contents of *N. sativa* seed extract and nanosuspension were determined using the aluminum chloride colorimetric method (Siddique et al., 2010). The reaction mixture was prepared by adding the 9.5 µL of NaNO_2_ (5%), 156 µL of distilled water, test samples of 38 µL of each, and 19 µL of AlCl_3_ (10% solution) and incubated at 25°C for 5 min. The optical density (OD) was measured at 510 nm. Finally, total flavonoid contents in the extract and nanosuspension were calculated from the calibration curve of catechin.

#### 2.4.3 DPPH Radical Scavenging Assay

The antioxidant activity of N. sativa extract and nanosuspension was determined using the 2,2-diphenyl-1-picrylhydrazyl (DPPH) free radical scavenging method. The 250 μL of methanolic solution of DPPH (0.0004%) (0.1 mM) and 2.5 μL of test sample were mixed properly. Then, the resultant mixture was incubated in the darkness at 25°C for 30 min. The absorbance was observed at 517 nm. Butylated hydroxytoluene (BHT) was used as a standard (Hussain et al., 2021). The inhibition of DPPH radical by test samples was calculated as follows:

%DPPH Inhibition=(A Blank−A Sample/A Blank)×100

### 2.5 Antidiabetic Activity

#### 2.5.1 Antiglycation Activity

The capacity of the natural compounds in the *N. sativa* extract and nanosuspension to prevent the methyl glyoxal–mediated production of fluorescence in bovine serum albumin (BSA) was tested *in vitro* using the antiglycation assay. The reaction solution of 100 mg D-glucose and 10 mg bovine serum albumin (BSA) in 1 ml of 67 mM sodium phosphate buffer (pH 7.2) was maintained with the test samples at 37°C for 2 days. Then, the absorbance of 0.2 ml diluted reaction solution was measured using the spectrophotometer at 370 nm as the excitation wavelength and at 440 nm as the emission wavelength (BioTek, Winooski, VT, United States). The solution without D-glucose was employed as a control in order to get desired results. The chemical metformin was utilized as a reference compound (Matsuda et al., 2003). The % inhibition by test samples was calculated as follows:

$$\%\text{inhibition=}\left[A_{\left(\mathrm{440}\;\mathrm{nm}\right)}/A_{\left(\mathrm{370}\;\mathrm{nm}\right)}-A_{\left(\mathrm{440}\;\mathrm{nm}\right)}\right]\times 100$$

#### 2.5.2 α-amylase Inhibition Assay

The α-amylase activity of natural compounds in the *N. sativa* extract and nanosuspension was measured using the colorimetric method. In particular, 30 µL test of samples and standard acarbose were maintained at room temperature in 96 wells plate for 10 min, followed by dissolving the 10 µL of amylase solution in 0.02 M sodium phosphate buffer and maintained at pH 6.9, 0.5 mg/ml. Following preincubation, 40 µL of 1% starch solution was added into the reaction mixture and incubated for 30 min. Then, 20 μL of 1 M HCl was added to each well. After that, 75 µL of iodine solution was added carefully to each well and absorbance was measured at 630 nm against a blank (Unuofin et al., 2018). The % inhibition by test samples was calculated as follows:

%inhibition=1-A(control)/A(sample)×100

### 2.6 Hemolytic Assay

The hemolytic potential of *N. sativa* extract and nanosuspension was performed against human red blood cells (RBCs). First of all, a 3 ml blood sample was collected and centrifuged for 5 minutes at 8,000 rpm to separate out the plasma from the cellular part of the blood. The plasma was discarded, and the red blood cell pellet was washed three times with 5 ml cold, sterile isotonic phosphate buffer saline (PBS; pH 7.4) and centrifuged again at 8,000 rpm for 5 min to make a cell suspension in normal saline. On a hemocytometer, the washed RBCs were calculated, with the RBCs remaining at 7.068 × 10^8^ cells/ml. After that, 180 µL of diluted blood cell suspension and 20 µL of each test sample were transferred in Eppendorf tubes and kept at 37°C for 40 min. After 15 min, the tubes were agitated, cooled, and centrifuged for 6 min before collecting the supernatant (100 µL) and diluted with PBS (900 µL); after that, 200 µL of chilled tube contents were transferred into the sterile ELISA microtiter plate. Then, 0.1 percent triton X-100 was employed as a positive control, while phosphate buffer saline was used a negative control (Powell et al., 2000). The absorbance was measured at 576 nm using an Elisa reader (BioTek, Winooski, VT, United States). The percent hemolytic inhibition was calculated by the following formula:

%hemolysis=A(sample)−A(negative control)/A(positive control)−A(negative control)×100

### 2.7 Antimicrobial Activity of *N. sativa* Extract and Nanosuspension

#### 2.7.1 Microbial Biofilm Inhibition

The biofilm inhibition of *N. sativa* extract and nanosuspension was evaluated using the microtiter plate method. The culture plate was loaded with 100 μL of sterilized nutrient broth, 100 μL of test material, and 20 μL of bacterial culture. Only nutrient broth and bacteria are present in the negative control well. Ciprofloxacin was utilized as a positive control in this assay. The plates were covered and incubated at 37°C for 24 h. The contents of each well were removed after 24 h and washing of sterile plates was carefully performed with phosphate buffer saline (Maintained pH:7.5) to eliminate any planktonic microorganisms. After cleaning, each well was filled with 100 μL of 99.9% ethanol and incubated for 15 min. After incubation, each well was stained with 100 μL of crystal violet dye, and the plates were incubated for another 10 min. The excess stain was removed after incubation, and the plates were washed again with distilled water (100 μL) and 33% glacial acetic acid (100 μL). The plate’s absorbance was measured at 630 nm (Hussain et al., 2021).

% biofilm inhibition=[A(control)−A(sample)/A(control)]×100

Furthermore, microscopic slides were prepared for biofilm inhibitions estimation. For overnight inoculation, the glass slide was loaded with nutrient broth and injected with a loop full of a pure culture of microbial strains. The contents of each slide were decanted after 48 h of incubation at 37°C. Then, for 7 min, the slide was dyed with 2% crystal violet. The slides were then immersed in distilled water for 5 min. The presence of an adhering coating of colored substance on the surface of the glass slides indicated a favorable outcome. As a negative control, a slide containing only broth and inoculums was used in the experiment. Similarly, positive control slides containing nutrition broth, inoculums, and a conventional antibiotic, ciprofloxacin, were used in the test to suppress microbial biofilm inhibition. Under a microscope, the selected test samples were compared to the positive control to check if they inhibited the microbial biofilm (Patel et al., 2019).

#### 2.7.2 Agar Well Diffusion Assay

The agar well diffusion test was performed to assess the antibacterial activity of sample fragments. The fresh bacterial culture of a bacterial isolates was pipetted into a sterilized agar medium to create nutrient agar plates. The wells of 6 mm diameter were formed after solidification, and the sample portions (100 μL) were drooped into the wells of the microplate. Ciprofloxacin was utilized as a control in this assay. The zone of inhibitions was formed. The antibacterial activity was measured in millimeters (mm) of the inhibition zone (Gonelimali et al., 2018).

### 2.8 Structural Analysis

#### 2.8.1 High-Performance Liquid Chromatography

The dried and hydrolyzed extract of *N. sativa* seeds was used for analysis. Appropriate combinations were made by dissolving 0.5 g of dried material with 20 milliliters of ethanol containing 1 g/L of BHT. Then, 10 ml of 1 mol. Hydrochloric acid into the reaction mixture was gently mixed, and then sonification was performed for 15 min (Iqbal et al., 2018). The mixture was then refluxed for 2 hours at 90°C in a water bath. After this process, the mixture was subjected to HPLC and a 20 µL sample was injected, and a measurement was taken at 280-nm. The retention period of several chemicals was used to identify them. HPLC analysis was performed using the Flexar FX-20 HPLC system. C-18 (25 cm × 4.6 mm; diameter 5 µm) column was used. The composition of mobile phases (A: CAN 70 + 30 MeOH; B: dH_2_0 + 0.5% glacial acetic acid) was used with the flow rate of 1 ml/min.

#### 2.8.2 Fourier-Transform Infrared Spectroscopy

FTIR was performed to characterize the structural properties of *N. sativa* seed powder. The material was first bireduced in chloroauric solution. After that, it was centrifuged for 15 min at 10,000 rpm. The pellet was obtained and washed three times with 20 ml deionized water to remove any undesired protein/enzymes not bound to particles. The materials were then dried and crushed in a potassium bromide pellet mill. Agilent Cary 630 FTIR system was used to perform the analysis, which was conducted in a diffuse reflect-array mode with a resolution of 4 cm. A total of 512 scans were performed in order to obtain the requisite acceptable signal/noise ratio (Manju et al., 2016).

#### 2.8.3 Gas Chromatography

Gas chromatography was performed to identify the different chemical compounds based on mass spectral comparisons. The GC equipment was fitted with a microliter sample that was exposed to capillary analysis. A GC column (SP-2560, 100 m × 0.25 mm internal diameter) (Superclo Inc., 24,056, United States) and a mass selective detector (HP 5972; Agilent Technologies, Palo Alto, CA, United States) were used. The temperature in the oven was maintained at 100°C for 1.5 min before progressively increasing to 270°C at 5°C per minute and injecting a 1 µL sample for analysis. Then, 99.9% nitrogen gas was used as a carrier gas with a flow rate of 1 ml per minute. The temperature of the sample injected was kept at 250°C throughout the experiment, and the split ratio was 20. The ionization mass was measured at 70 eV. For 60 min, mass spectra in the range 40–600 m/z were obtained. The mass spectra of the chemicals were compared to identify the different chemical compounds. Elution through the column was noticed in electrical signals as the chemical separated and resulted in the m/z value that was carefully calibrated by using the mass spectrum graph that represented the peaks of different chemical compounds. In the mass spectrum graph, different chemical compounds were compared and identified on the basis of mass spectra with already existing library (Nisar et al., 2019).

### 2.9 Statistical Analysis

Data were analyzed by analysis of variance (ANOVA) for the comparison between means of two populations simultaneously and to test the significance of formulation parameters with the level of p significant at < 0.05. Data were finally expressed as mean, standard deviation, or percentage (%) of triplicate measurements.

## 3 Results and Discussion

Medicinal plants are a rich source of active biological compounds such as flavonoids, tannins, nitric acid, polyphenols, and phenols (Priya et al., 2017). This research was aimed at finding the pharmacological properties and bioavailability of bioactive compounds that are present in *N. sativa* nanosuspension and its extract.

### 3.1 *In vitro* Antioxidant Potential of Extract and Nanosuspension

*In vitro* antioxidant activities of *N. sativa* extract and nanosuspension are presented in Table 1 **.** Nanosuspension contains 478.63 ± 5.00 mg GAE/100 g as compared to the extract that contains only 326.7070 ± 4.38 mg GAE/100 g phenolic content. Flavonoid contents also showed the same reciprocal results as phenolic contents. Nanosuspension contains 192.23 ± 1.390 mg CE/100 g, while the extract contains only 104.26 ± 2.23 mg CE/100 g total flavonoids. The results also indicated that the nanosuspension of *N. sativa* showed higher antioxidant potential than the *N. sativa* extract. This current study is in line with the previous study having 4.07 ± 2.68% radical scavenging activity found using the DPPH assay for its extract as compared to nanosuspension showed 16.74 ± 1.88%, which can show the bioavailability of the compounds for a long time in nanosuspension. Statistical analysis revealed that TPC, TFC, and DPPH assays showed highly significant differences (*p* < 0.01).

**TABLE 1:** Comparison of antioxidant potential of nanosuspension of *N. sativa* with control.

Treatments	TPC (mg GAE/100 g)	TFC (mg CE/100 g)	DPPH scavenging potential (%)
NS	478.63 ± 5.00	192.23 ± 1.390	16.74 ± 1.88
E	326.7070 ± 4.38	104.26 ± 2.23	4.07 ± 2.68
Control	750.87 ± 6.63	244.44 ± 2.63	90 ± 0.00

a NS: *Nigella sativa* nanosuspension, E: *Nigella sativa* extract.

Our results are also aligned well with the previous studies. Chahardehi et al. (2009) and Hussain et al. (2021) also described that the Folin–Ciocalteu method was the highest optimized method for the investigation of total phenolic contents. Another study by Dalli et al. (2021a) investigated that total phenolic and flavonoid contents present in *N. sativa* seeds and their antioxidant properties have been a subject of rigorous experimentation and many have experimented with different solvents and different extraction techniques as well. This study also confirms the findings of Tunç et al. (2020).

The DPPH scavenging assay was performed by the protocols that are already documented by Hussain et al. (2021). Naturally occurring phenols are more effective than vitamins (Yasmeen and Hassnain, 2016). Previous studies experimented with different extraction techniques, and extracts of *N. sativa* seeds were subjected to the DPPH scavenging assay; and the result they obtained was 61.7% (Dalli et al., 2021a). They also have significant amounts of other antioxidants such as different tocopherol and tocotrienol isomers are present in the alpha, beta, gamma, and delta forms along with these compounds extract that also contain β-sitosterol, respectively. Another study also concluded that the methanolic extract of *N. sativa* seeds showed less percentage of the DPPH inhibition assay than the ethanolic extract, and the methanolic extract only inhibits 3.77% of the DPPH scavenging assay (Vahitha et al., 2019).

Our results are also highly aligned with the previous studies as Arif et al. (2021) revealed that nanosuspensions of *N. sativa* extracts showed a maximum of up to 55% free radical scavenging activity at 1,000 mg/ml concentrations while the lowest activity was up to 28% at 250 mg/ml. This study showed that increasing the concentrations of nanosuspensions and *N. sativa* extracts significantly increased the DPPH free radical scavenging activity. Another study conducted by Veeramani et al. (2022) also investigated that nanosuspensions of *N. Sativa* extract exhibited a maximum free radical scavenging activity at 500 g/ml. Thus, increasing the concentrations of nanosuspensions of *N. sativa* extracts significantly increased the DPPH free radical scavenging activity.

### 3.2 Antidiabetic Potential of Extract and Nanosuspension

The results of the antidiabetic potential of the extract and nanosuspension of *N. sativa* are presented in Table 2. The results indicate that nanosuspension showed higher antiglycation activity than extract and referral control metformin. Nanosuspension showed 58 ± 0.912% antiglycation, while extract and metformin showed 54 ± 2.165% and 56.9 ± 2.16% antiglycation activity, respectively. Nanosuspension and extract showed 18.0 ± 1.367% and 12.9 ± 2.965% inhibition against bacterial alpha-amylase, respectively. Furthermore, nanosuspension and extract showed 17.8 ± 1.645% and 17.2 ± 1.895% inhibition against fungal alpha-amylase, respectively (Table 2). Statistical analysis revealed that antiglycation and alpha-amylase inhibition assays showed a significant difference (*p* < 0.05).

**TABLE 2:** Antidiabetic potential of extract and nanosuspension of *N. sativa*.

Treatments	% Antiglycation activity	% alpha-amylase inhibition	
	Bacterial Fungal		
NS	58 ± 0.912,133	18.0 ± 1.3675	17.8 ± 1.6445
E	54 ± 2.16547	12.9 ± 2.9655	17.2 ± 1.8957
Metformin/Acarbose	56.91 ± 2.162	82.53 ± 2.6445	82.53 ± 2.6445

a NS: *Nigella sativa* nanosuspension, E: *Nigella sativa* extract.

Diabetes mellitus (DM), a prevalent incurable metabolic disorder, is a worldwide health hazard (Vijayakumar et al., 2021). Consistent hyperglycemia can cause chronic micro and macrovascular effects in people with diabetes, including cardiovascular disease, retinopathy, neuropathy, and stroke (Jiang, 2017). Several medicines are now available in the market to treat DM problems. However, certain common medications, such as inhibitors of alpha-glucosidase like acarbose, sulfonylureas, glinides, biguanides, and miglitol, can induce adverse effects, including liver problems, nausea, flatulence, abdominal discomfort, renal tumors, hepatic damage, dark urine, and low blood glucose (Gharibi et al., 2013). To replace these synthetic medicines, novel antioxidant and antidiabetic medicines derived from traditional plants are necessary. Phytochemicals (particularly polyphenols) can help control oxidation and stress-related persistent illnesses, including cardiovascular disease and diabetes (Kwon et al., 2008).

The present study also correlates with the study conducted by Dalli et al. (2021b) that showed 66.3% alpha-amylase inhibition by n-hexane extract fraction of *N. sativa*. Another study conducted by Rani et al. (2018) showed that in diabetic rats, the oral treatment of an integrated nanoformulation for 21 days reduced fasting blood glucose level. Another recent study conducted by Vijayakumar et al. (2021) showed that α-amylase inhibition plays a critical role in the prevention of increased blood glucose levels. Our study indicated that nanosuspension contains higher concentrations of phenolic compounds than the extract fraction of seeds. So, it was assumed that if the bioavailability of phenolic compounds decreases subsequently, α-amylase inhibitory activity can be decreased significantly. Thus, ethanolic extract fractions can be used for controlling hyperglycemia. Further *in vitro* studies showed the insights of the possible key enzyme inhibitory effects related to diabetes mellitus, which is in accordance with our studies. These inhibitory effects can be attributed to the antioxidant activity and phenolic contents of the medicinal plants (Yarrappagaari et al., 2020).

Our results are also in close agreement with the previous studies. Veeramani et al. (2022) revealed that the nanosuspensions of *N. sativa* extracts showed maximum enzyme inhibition activity of alpha-amylase at the highest concentration (500 g/ml), thus exhibiting a maximum antidiabetic property of 81%, respectively. The presence of phytochemicals such as flavonoids and polyphenols might be the explanation for Au-NPs’ maximal amylase inhibitory property due to their capacity to decrease oxidative stress which qualifies them as an antioxidant, while their ability to inhibit carbohydrate hydrolyzing enzymes qualifies them as an antidiabetic agent. Because the Au-NPs are made by phytoconstituents having high antioxidant and antidiabetic properties, they might be exploited as a promising ingredient in antidiabetic medications in the future.

Vijayakumar et al. (2021) investigated the antidiabetic properties of *N. sativa* seed extract covered with silver nanoparticles known as Bc-AgNPs. The study’s findings suggested that NSE-derived AgNPs (BCAgNPs) from medicinal plants might be used for controlling the severity of diabetes mellitus, inflammatory diseases, and microbe-related illnesses in the future. Nanosuspension was created to improve its bioavailability. However, no studies on the antihyperglycemic efficacy of prepared nanosuspensions have been published so far. As a result, the goal of this research was to demonstrate the improvement in the bioavailability of bioactive compounds for treatment purposes.

### 3.3 Antibacterial Potential of Extract and Nanosuspension

**FIGURE 2:**
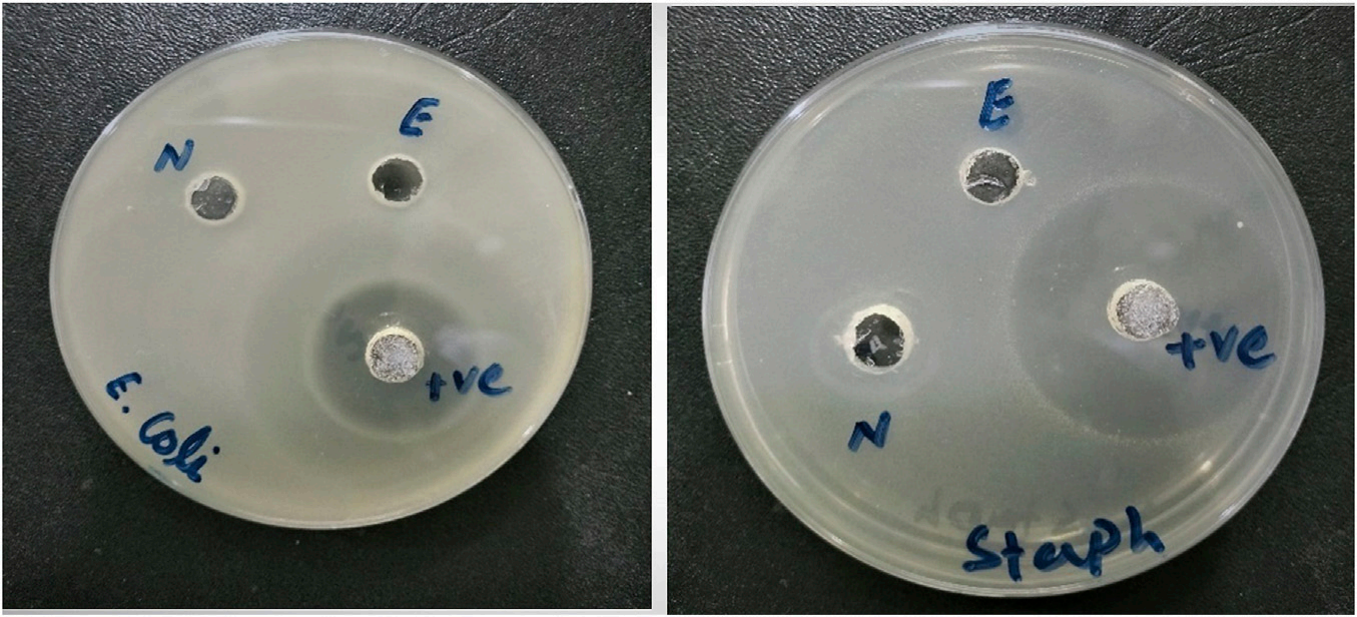
Antibacterial activity of *N. sativa* extract and nanosuspension against *Escherichia coli* and *Staphylococcus aureus* isolates.

**FIGURE 3:**
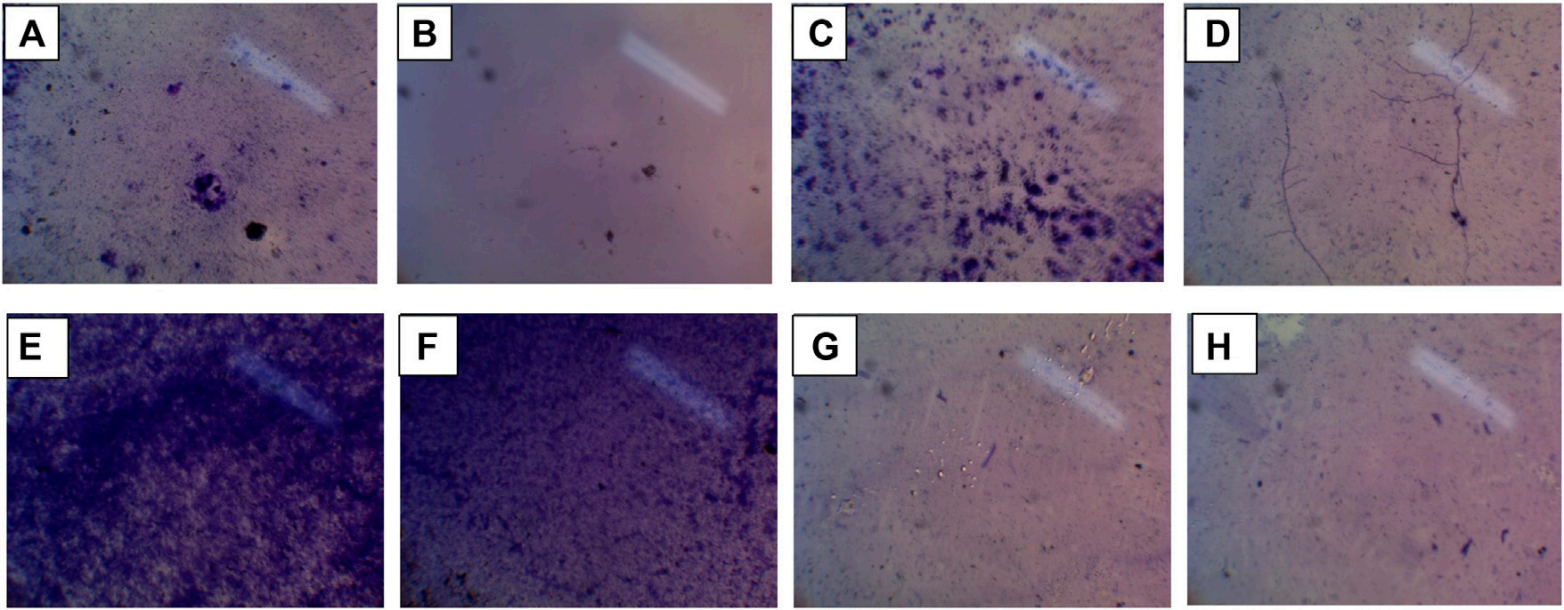
Phase-contrast microscopy of biofilm inhibition **(A)** (Qualitative assay) inhibition of *E. coli* by extract fraction (min.), **(B)** inhibition of *E. coli* by nanosuspension fraction (max.), **(C)** inhibition of *S. aureus* by extract fraction (min.), **(D)** inhibition of *S. aureus* by nanosuspension fraction (max.), **(E)** inhibition of negative control against *E. coli*, **(F)** inhibition of negative control against *S. aureus*, **(G)** inhibition of positive control against *E. coli*, and **(H)** inhibition of positive control against *S. aureus.*

The extract and nanosuspension of *N. sativa* were tested for investigating the antibacterial activity against different pathogens such as *Escherichia coli* and *Staphylococcus aureus*. Antibacterial activity was performed using the agar well diffusion method is a qualitative approach commonly used to test plant extracts for antibacterial activity. The results of the antibacterial activity of the extract and nanosuspension of *N. sativa* are presented in Table 3 and Figure 2. The extract and nanosuspension showed significant antibacterial activities against these isolates. But nanosuspension showed higher antibacterial activities than the extract. Even against *E. coli*, nanosuspension showed higher biofilm inhibition (66.44 ± 3.529%) than the positive control (ciprofloxacin) (59.39 ± 3.013%) and extract (44.96 ± 2.238%). Moreover, nanosuspension and extract showed higher biofilm inhibition against *E. coli* as compared to *S. aureus*. Against *S. aureus*, nanosuspension and extract showed lower biofilm inhibition as compared to a positive control (Ciprofloxacin) 27.73 ± 1.523% and 9.24 ± 0.862%, respectively. The results from phase-contrast microscopy showed the morphological features and biofilm inhibition and destructive potential of *N. sativa* extract and nanosuspension on biofilms of *Escherichia coli* and *Staphylococcus aureus* isolates (Figure 3). As inhibition flows in the following realm NS_inb_ *E. coli* < PC_inb_ *E. coli* < E_inb_ *E. coli* < PC_inb_ *S. aureus* < NS_inb_ *S. aureus* < E_nb_ *S. aureus*. The negative control against both strains showed nil or relatively negligible inhibition. In the well diffusion method, the extract and nanosuspension showed no activity against *E. coli*, but nanosuspension showed a significant zone of inhibitions against *S. aureus.* Statistical analysis revealed that the agar well diffusion method and biofilm inhibition method showed highly significant differences (*p* < 0.01).

**TABLE 3:** Antibacterial activity of *N. sativa* extract and nanosuspension against *Escherichia coli* and *Staphylococcus aureus* isolates.

Treatments	% Biofilm inhibition		Zone of inhibition (mm)	
	*Escherichia coli*	*Staphylococcus aureus*	*Escherichia coli*	*Staphylococcus aureus*
NS	66.44 ± 3.529	27.73 ± 1.523	00	00
E	44.96 ± 2.238	9.24 ± 0.862	00	15 ± 0.984
Ciprofloxacin	59.39 ± 3.013	42.01 ± 2.862	26 ± 1.239	39 ± 1.976

a NS: *Nigella sativa* nanosuspension, E: *Nigella sativa* extract.

Topcagic et al. (2017) conducted research on diethyl extract of *N. sativa* seeds with various concentrations to assess the inhibitory capability of several microorganisms. Moreover, methanolic and chloroform extracts have shown inhibitory action for controlling the different infections caused by *S. aureus* and *H. pylori*. *N. sativa* seeds have been proved efficient against both Gram-positive and Gram-negative bacteria. Another recent study by Almatroudi et al. (2020) to access the antimicrobial activity and biofilm suppression were also observed in the biologically produced particles against *E. faecalis*, *E. coli*, *S. aureus*, *K. pneumoniae*, and *P. aeruginosa*. Even silver NPs (4–17 nm) and gold NPs (12–20 nm) have previously been synthesized using the extract from this plant. Furthermore, the biogenic nanoparticles’ biofilm inhibitory properties are being investigated against pathogens such as *Pseudomonas aeruginosa*, *Listeria monocytogenes*, *Chromobacterium violaceum*, and *E. coli* and for possible use as a food packing material and preservative. In addition, treatment with sub-repressive dosages of NS-ZnNPs resulted in a considerable reduction in preformed biofilms in all bacterial strains examined. According to the study, the synthesized ZnNSs might be used as a QS and biofilm inhibitor that could be used not only as an antipathogenic but also as a nontoxic bioactive material for food packaging and food preservation (Al-Shabib et al., 2016). Both the agar well diffusion method and biofilm inhibition method supported our results.

Mahfouz et al. (2020) revealed that higher concentrations of the *N. sativa* AgNPs determine the growth inhibition of different bacterial strains. In that study, *N. sativa* extract (NSE) was tested against four bacterial strains and showed maximum antibacterial activity against *Staphylococcus aureus*, *Escherichia coli*, and *Bacillus subtilis*, with zones of inhibitions of 7.03 ± 0.058, 10 ± 0.000, and 9 ± 0.0000, respectively. Our results are also aligned with the previous studies as Alkhathlan et al. (2020) revealed that nanosuspensions of *N. Sativa* extracts showed the higher concentrations of AgNPs and determine the growth inhibition of different bacterial strains. *N. sativa* extract (NSE) was tested against four bacterial strains and showed maximum antibacterial activity at (50–500 g/ml) including Gram-negative (*Escherichia coli* and *Pseudomonas aeruginosa*) and Gram-positive (*Staphylococcus aureus* and *Bacillus subtilis*).

Due to higher antibacterial resistance, the antibacterial activity of plants proves as a safer alternation of antibiotics (Mukhtar et al., 2019). Due to the presence of thymoquinone and other bioactive compounds in the test samples, we get antibacterial activity. But as compared to extract, nanosuspension showed higher antibacterial activity due to the enhanced bioavailability of these bioactive compounds.

### 3.4 Hemolytic Activity of Extract and Nanosuspension

Nanosuspension showed higher hemolytic activity than the extract as 7.8 ± 0.1% and 6.5 ± 0.3%, respectively, but showed lower hemolytic activity than the positive control, which showed maximum hemolytic activity of 96.45 ± 0.00% (Table 4). Statistical analysis revealed that the hemolytic assay showed a highly significant difference (*p* < 0.01). This assay was performed with RBCs. A measure of 0.1% of Triton X-100 was taken as a positive control. Phosphate buffer saline was used as a negative control.

**TABLE 4:** Hemolytic activity of *N. sativa* extract and nanosuspension.

Treatments	% Hemolysis
NS	7.8 ± 0.1
E	6.5 ± 0.3
Triton X-100	96.45 ± 0.00

a NS: *Nigella sativa* nanosuspension, E: *Nigella sativa* extract.

*N. sativa* herbal extract loaded silk nanofibrous mat formed to evaluate its biomedical importance, this study evaluated that the nanofiber showed higher hemolytic activity than its simultaneous concentrated extract (Muthumanickkam et al., 2020). Due to the morphological and physiological characteristics of RBCs, they are extensively studied, mainly in drug development and discovery (Shabbir et al., 2013).

Another study found that raising the formulation percentages (v/v) improved the hemolytic activity of thymoquinone-loaded cubosomal formulations. Our results are also aligned with the previous studies. Mehanna et al. (2020) revealed that nanosuspensions of *N. sativa* extracts exhibited maximum hemolytic activity of 30 ± 0.90% in the presence of Triton X100 solution (2%).

Desai et al. (2020) furthermore described that *N. sativa* suspensions of PtNPs showed antihemolytic activity at concentrations of 20–100 mg/ml up to 45–50%. They also found that PtNPs showed maximum inhibition up to 51% at 20 mg/ml.

### 3.5 Structural Characterization

The HPLC and GC were used for the phytochemical screening. Phenolic compounds such as gallic acid were identified by the HPLC. At the same time, benzoic acids and ethers are identified by GC. The metabolism of phenolic compounds varies from organism to organism. The derivatives of benzoic acid and cinnamic acid are commonly two families of phenolic acids that are considered present in plants (Topcagic et al., 2017).

#### 3.5.1 High-Performance Liquid Chromatography

**FIGURE 4:**
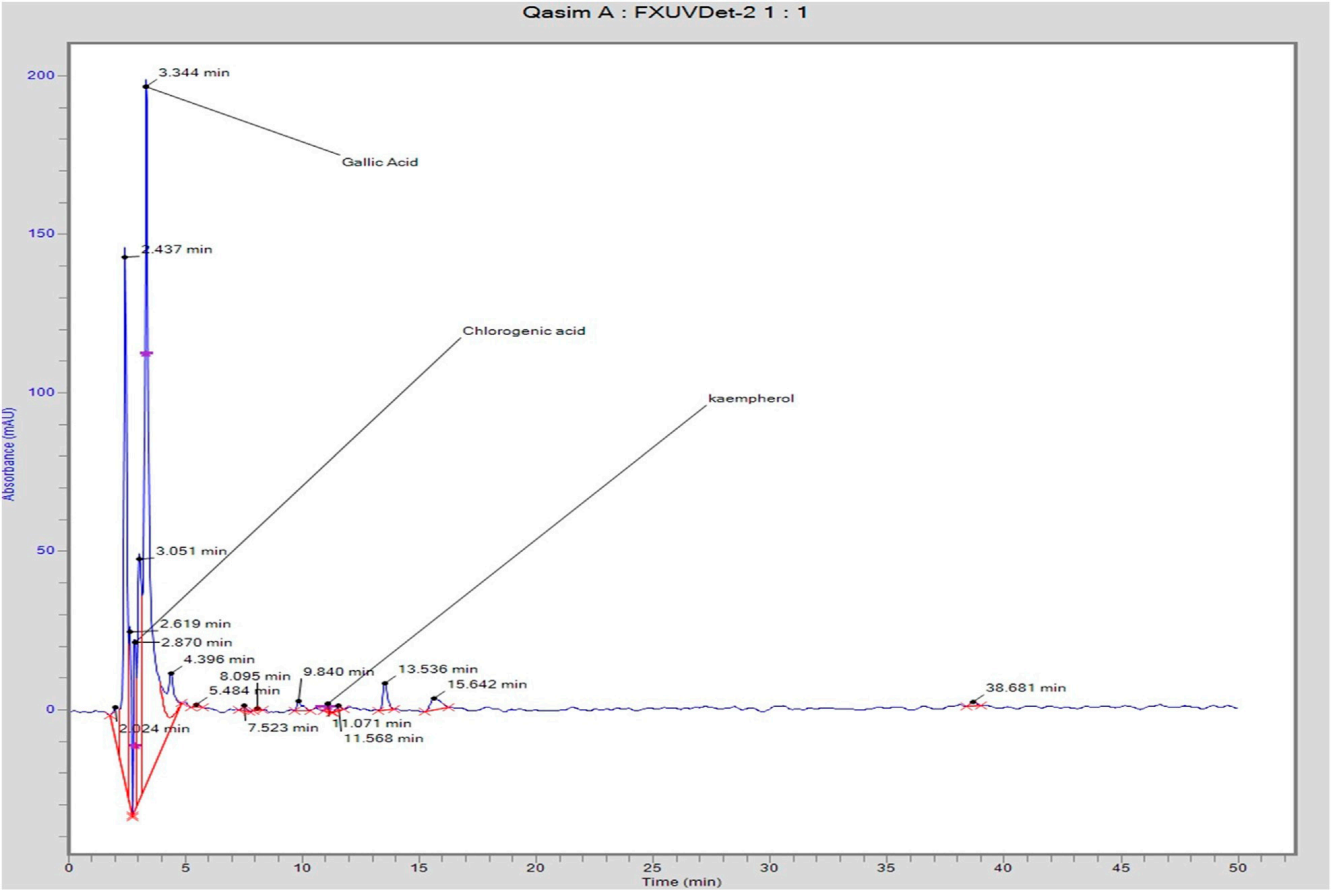
HPLC profile of *N. sativa* seed extract.

The Flexar FX-20 HPLC system was used to evaluate and identify phenolics at room temperature, as shown in Figure 4 and Table 5. The chromatogram generated by the HPLC showed peaks of different bioactive compounds present in the sample according to their retention time in the column. According to this chromatogram, the retention time and other details about HPLC results are shown in Table 5. Table showed the summarized data of bioactive compounds identified and quantified by HPLC analysis. It concluded that *Nigella sativa* ethanolic seed extract contains chlorogenic acid, gallic acid, and kaempferol in varying quantities at retention times of 2.870, 3.344, and 11.0701 min, respectively.

**TABLE 5:** Quantification of different bioactive compounds.

Retention	Compound	Area (mv. s)	Height	Amount (ppm)
Time (min)	Name			
2.870	Chlorogenic acid	373,556.6	53,080.9	4.22
3.344	Gallic acid	3,832,983.8	220,651.2	3.34
11.071	Kaempferol	23,063.8	52,511.9	0.09

Polyphenols are the compounds responsible for the protection of cells from biological oxidative stress and improving their life. The difference in the results of different studies in the past and the current study is possibly due to the difference of solvent used as well as the identification methods used. A previous study reported the presence of quercetin, isoquercetin, rutin, and glucuronide in the seed extract of *N. sativa*, but they did not report the quantitative study of phenolic and flavonoid compounds found in the seeds. This study is coherent and in line with the previous study (Dalli et al., 2021b). High-performance liquid chromatography is the chief analytical technique used extensively in the biological laboratories for the identification and quantification of various biologically active functional groups present in the test sample.

Alshwyeh et al. (2022) revealed the different formulations of *N. sativa* extracts exhibited the volatile compounds, namely, n-hexadecanoic acid (34.40%), hexadecanoic acid ethyl ester (35%), oleic acid (38%), ethyl oleate (39%), and hexadecenoic acid (35%). Tiji et al. (2021) revealed that the qualitative investigation of ethanolic extract of *N. sativa* seeds by HPLC showed the presence of gallic acid, hydroquinone, apigenin, naringenin, ascorbic acid, cysteine, rutin, quercetin, and kaempferol.

#### 3.5.2 Fourier-Transform Infrared Spectroscopy

**FIGURE 5:**
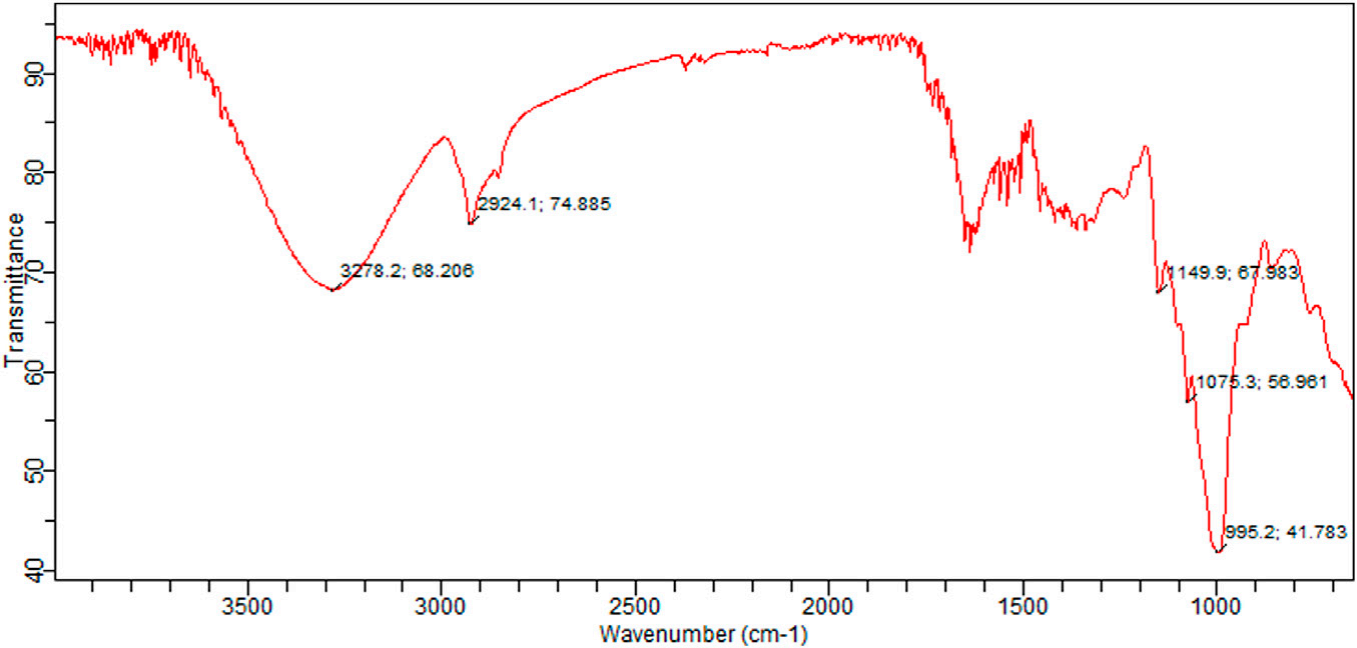
FTIR spectra of *N*. *sativa* seed powder.

The interferogram generated by FTIR is the graphical representation of compounds identified in the powder of *N. sativa* seeds (Figure 5). Table 6 showed the absorption values as predicted by the FTIR which identified different functional groups present in the black seeds. A strong peak at 3,278 cm^−1^ indicated the presence of alcohols. A band obtained at 1,924 cm^−1^ indicated the presence of alkane in the sample. The band at 1,149 cm^−1^ corresponds to the presence of alkyl halide. Two medium bands at 1,075 cm^−1^ and 995 cm^−1^ also identified the presence of alkyl halide.

**TABLE 6:** FTIR spectrum chart indicating the identified functional groups in *N. sativa* seed powder.

Peak no	Characteristic absorption	Identified functional groups	Compound class
1	3,278	O–H stretching	Alcohol
2	2,924	C–H stretching	Alkane
3	1,149	C–F stretching	Alkyl halide
4	1,075	C–F stretching	Alkyl halide
5	995	C–F stretching	Alkyl halide

Fourier-transform infrared is the most suitable and quick method used for the identification of different functional groups present in a sample under examination and based on those functional groups, it concludes the structure of sample material. Thus, it is most commonly used for the identification of organic compounds such as acids, polyphenols, and many other known as well as unknown moieties present in the sample. In a recent investigation, the FTIR spectra of *N. sativa* essential oil revealed high absorption bands at 3,378 and 2,848 cm^−1^, which correspond to polyphenol O–H and C–H stretching. The C=O stretching of alkanes correlates to the absorption peak at 1,694 cm^−1^. The C–OH group of amides is represented by the peak at 1,604 cm^−1^. The stretching vibrations of aromatic rings are represented by the band at 1,380, 1,118, and 1,040 cm^−1^ (Manju et al., 2016).

Our results are also aligned with the previous studies. Veeramani et al. (2022) revealed that FTIR analysis of *N. Sativa* extracts, the spectra of *N. sativa* aqueous seed extract exhibit strong bands at 1,027 cm^−1^ and 1,035 cm^−1^, 1,633 cm^−1^ and 1,648 cm^−1^, 2,925 cm^−1^ and 2,927 cm^−1^, 3,253 cm^−1^ and 3,275 cm^−1^, and 2,925 cm^−1^ and 2,927 cm^−1^, respectively. C–H bond stretching in alkyl groups was shown by the bands at 1,027 cm^−1^ and 1,035 cm^−1^. CO– stretching acid groups were responsible for the absorption peaks at 1,633 cm^−1^ and 1,648 cm^−1^. The CH–stretching of the alkane functional group caused the strong bands at 2,925 cm^−1^ and 2,927 cm^−1^. The NH stretching of the amine group caused the broadening of peaks at 3,253 cm^−1^ and 3,275 cm^−1^. The presence of amine linkage showed that the seed extract reaction mixture included flavonoids or polyphenols.

#### 3.5.3 Gas Chromatography

Phytochemical screening was performed by gas chromatography that showed the presence of biomolecules and different chemical compounds in the ethanolic extract. Table 7 shows the presence of carbohydrates, oil contents, different types of amino acids and proteins, flavonoids, steroids, and alkaloids. As described in a previous study, gas chromatography–mass spectrometry (GC–MS) analysis of *N. sativa* revealed the presence of the ß-pinene, D-glucose, 6-O-α-D-galactopyranosyl, O-cymene, DL-arabinose, trans-4-methoxy, and vice versa (Hadi et al., 2016). Furthermore, bioactive chemicals constituting roughly 85 percent of the total quantity of volatile oil of *N. sativa* were identified using gas chromatography and gas chromatography–mass spectrometry analyses. The main identified compounds were p-cymene (36.2%), thymoquinone (11.2%), and α-thujene (10.0%) (Ramadan, 2016). The presence of compounds in *N. sativa* seed extracts also depends upon the extraction techniques and pretreatment of the sample that is characterized by the GC. The used GC column also played an important role in the characterization of the compounds present in the sample.

**TABLE 7:** Identified compounds by GC in *N. sativa* ethanolic extract.

Sr.no	Name of the compound	Retention time (min)	Percentage (%)	Molecular formula
1	Thymoquinone	15.324	7.568	C_10_H_12_O_2_
2	Amyl benzene	12.064	0.280	C_11_H_16_
3	Carvacrol	16.467	3.110	C_10_H_14_O
4	Butyl benzene	9.678	0.381	C_10_H_14_
5	Alpha-ylangene	16.780	0.048	C_15_H_24_
6	Delta-cadinol	16.987	0.0392	C_15_H_26_O
7	1-Monolinolein	24.44	3.402	C_21_H_38_O_4_
8	Myristic acid	20.467	0.142	C_14_H_28_O_2_
9	1,13 Tetradecadiene-3-1	14.467	0.04	C_14_H_24_O
10	Trilinolein	23.02	21.324	C_57_H_98_O_6_
11	Laevojunenol	19.021	0.112	C_15_H_26_O
12	O-eugenol	16.689	1.274	C_10_H_12_O_2_
13	Beta caryophyllene	16.128	0.187	C_15_H_24_
14	Acetoiso vanillone	17.346	5.346	C_9_H_10_O_3_
15	Hexa deca methyl cyclo	19.23	0.086	C_16_H_48_O_8_Si_8_
	Octa siloxane			
16	Cycloocta siloxane hexa deca methyl	18.789	0.098	C_16_H_48_O_8_Si_8_
17	Monoelaidin	22.42	3.023	C_21_H_40_O_4_
18	Laevojunenol	19.021	0.112	C_15_H_26_O
—	Total	—	49.28%	—

Another study conducted by Vijayakumar et al. (2021) revealed that the GC–MS analysis of *N. sativa* seed extract showed the presence of 16 major peaks, and the components (relative content) corresponding to the fractions represented by the peaks have been determined as follows: octadecene (3.175%), 1,3-cyclohexadiene (3.735%), methyl-benzene (4.405%), styrene (6.485%), cyclohexene, 3-methylene-4-vinyl (6.931%), 1,3,6-octatriene (7.082%), tetramethylphenol (12.872%), acetophenone (16.147%), lauric acid (16.868%), 2-pentenoic acid (17.594%), oxalic acid (17.656%), 2-methyl-4-penten-2-ol (17.812%), 9,12,15-octadecatrienoic acid (19.296%), pentadecanoic acid (20.100%), 2-naphthalenecarbonitrile (21.361%), and hexadecanoic acid (22.326%).

Our results are also aligned with another previous study. Farhan et al. (2021) revealed that the nanosuspensions of *N. sativa* extracts exhibited the volatile compounds longifolene (3%), p-cymene (31.50%), a-thujene (9%), and thymoquinone (25.35%). Due to the high volatile nature of compounds in nanosuspensions of N. Sativa extracts, they can be used for therapeutic purposes, especially in pharmaceutical industries for drug discovery.

## 4 Conclusion

The objective of the present study was to apply the nanotechnology approach for exploring the enhanced bioactivities of freshly prepared *Nigella sativa* L nanosuspensions and phytochemical profiling of *N. sativa* seed ethanolic extract. The *N. sativa* L nanosuspension was formalized in a cost-effective manner. The characterization analyses were performed by High-performance liquid chromatography (HPLC), Fourier-transform infrared spectroscopy (FT-IR), and Gas chromatography (GC). HPLC chromatogram revealed the presence of chlorogenic acid, gallic acid, and kaempferol in varying quantities at retention times 2.870, 3.344, and 11.0701 min, respectively. The nanosuspension of *N. sativa* seeds showed higher total phenolic (478.63 ± 5.00 mg GAE/100 g) and total flavonoid contents (192.23 ± 1.390 mg CE/100 g) than the ethanolic seed extract which were confirmed using Folin–Ciocalteu and calorimetric methods. In addition, antioxidant activity was performed using the DPPH free radical assay, and nanosuspension showed higher potential to scavenge the free radicals (16.74 ± 1.88%) than the extract. The antidiabetic activity was performed by antiglycation and α-amylase inhibition assays, nanosuspension showed higher antidiabetic potential (antiglycation (58 ± 0.912%)) and (bacterial α-amylase inhibition (18.0 ± 1.3675%)), respectively. Antibacterial activities were confirmed using the well diffusion method, and biofilm inhibition assay. Nanosuspensions showed higher biofilm inhibition activity against *Escherichia coli* (66.44 ± 3.529%) than the extract (44.96 ± 2.238%) and ciprofloxacin (59.39 ± 3.013%). Hemolytic activity was performed and nanosuspension showed higher hemolytic activity than the extract as 7.8 ± 0.1% and 6.5 ± 0.3%, respectively. It was concluded that nanosuspension of *N. sativa* L. in our study possesses excellent enhanced bioactivities and high bioavailabilities than its ethanolic extract and can be recommended for therapeutic applications. These cost-effective nanoformulations could serve as a platform for the development of combination protocols for the characterization of liquid state nanosuspensions and future *in vitro* studies. This study also led to the foundation for plant-based nanosuspension synthesis by replacing the synthetic formulations in an adequate manner.

## Data Availability

The original contributions presented in the study are included in the article/Supplementary Material, further inquiries can be directed to the corresponding authors.
